# Primordial soup was edible: abiotically produced Miller-Urey mixture supports bacterial growth

**DOI:** 10.1038/srep14338

**Published:** 2015-09-28

**Authors:** Xueshu Xie, Daniel Backman, Albert T. Lebedev, Viatcheslav B. Artaev, Liying Jiang, Leopold L. Ilag, Roman A. Zubarev

**Affiliations:** 1 Division of Physiological Chemistry I, Department of Medical Biochemistry and Biophysics, Karolinska Institutet, Scheelesväg 2, SE-17 177 Stockholm, Sweden; 2 Department of Organic Chemistry, Moscow State M. V. Lomonosov University, 119991, Moscow, Russia; 3 LECO Corporation, 3000 Lakeview Avenue, St Joseph, MI, USA; 4 Department of Environmental Science and Analytical Chemistry, University of Stockholm, Stockholm, Sweden

## Abstract

Sixty years after the seminal Miller-Urey experiment that abiotically produced a mixture of racemized amino acids, we provide a definite proof that this primordial soup, when properly cooked, was edible for primitive organisms. Direct admixture of even small amounts of Miller-Urey mixture strongly inhibits *E. coli* bacteria growth due to the toxicity of abundant components, such as cyanides. However, these toxic compounds are both volatile and extremely reactive, while bacteria are highly capable of adaptation. Consequently, after bacterial adaptation to a mixture of the two most abundant abiotic amino acids, glycine and racemized alanine, dried and reconstituted MU soup was found to support bacterial growth and even accelerate it compared to a simple mixture of the two amino acids. Therefore, primordial Miller-Urey soup was perfectly suitable as a growth media for early life forms.

Abiogenesis theory pioneered by Alexandr Oparin (1894–1980) postulates that under proper conditions life can arise spontaneously from non-living molecules. Oparin’s book, *The Origin of Life*, was first published in 1924 and quickly became a worldwide bestseller^1^. Oparin speculated that life has emerged through random processes in ‘a biochemical soup’ that once existed in the oceans. According to that theory, spontaneous origination of life requires the presence of the correct mix of chemicals and free energy. The organic molecules necessary for life have been created in the atmosphere of early Earth by such forces as lightning, electric discharges from the sun wind, ultraviolet light and meteorites. These molecules rained from atmosphere into the primitive oceans, where the free energy necessary for life self-organization was supplied by deep-sea hydrothermal vents, hot springs, volcanoes, and earthquakes. Over the decades after Oparin’s book was published, Haldane^2^, Bernal^3^, Calvin^4^ and Urey^5^ tried to produce evidence supporting this scenario. The critical breakthrough came in 1953 from the experiment performed by Stanley Miller (1930–2007), a graduate student of Harold Urey (1893–1981). Following the instruction of Urey who believed at a time that the early atmosphere was reducing and contained large amounts of methane^6^, Miller introduced water as well as a mixture of hydrogen, methane and ammonia gases in a closed apparatus, and then subjected this mixture to a high-voltage (60 kV) electric discharge for a week, while the water was simultaneously heated. The products accumulated in a water trap below a water-cooled condenser^7^. Since 1953, this experiment has been repeated hundreds of times in dozens of labs. There now are even video instructions available how to construct and run the apparatus^8^.

The experiment usually progresses as follows. Within a day or two of sparking and heating, a pink stain is formed on the sides of the discharge flask, and the water in the trap acquires yellowish color. After several days, the stain turns dark red, and then turbid, while the trap water changes color from yellow to dark brown. At the end, the primary substances in the gaseous phase become carbon monoxide CO and nitrogen N_2_. The dominant material in the water trap is a complex mixture of organic molecules, including aldehydes and cyanides, as well as ‘tar’, mostly insoluble in water^9^. The simplest biologically useful amino acids are also present — mostly glycine and alanine, that together compose 1–3% of the solid residue, as well as smaller amounts of other biological amino acids^10^. Both L- and D- chiral forms of amino acids are produced in roughly equal amounts (racemic mixture), even though a recent report suggests that biologically relevant L-forms may be somewhat more abundant^11^.

Miller-Urey experiment marked the beginning of a new scientific field - prebiotic chemistry^12^; it is now the most commonly cited evidence for abiogenesis in science textbooks^13^. Yet sixty years after this seminal experiment, the debate is still ongoing whether it represents a definite argument for spontaneous life emergence, or even whether the brown soup produced in this experiment can support life^14^. It has been argued that, besides highly toxic for some organisms gaseous CO, as well as aldehydes and cyanides in solution, the Miller-Urey mixture (MU mixture) contains D-amino acids that, according to some research^15^,^16^, can also be toxic to biological organisms^14^.

Some of the above argumentation can be easily refuted. For instance, for methanogenic archaea, carbon monoxide is a nutrient. Furthermore, Kun and Somerville have obtained in 1971 a strain of *Escherichia coli* that could metabolize several D-amino acids, including D-alanine and D-glutamic acid^17^. But in general, there has been a surprising lack of experimental proof that the MU mixture produced from simple gases can indeed support life of primitive organisms. Here we attempted to fill this gap.

## Methods

### Miller-Urey experiment

Methane (CH_4_), hydrogen (H_2_), and ammonia (NH_3_) gases were purchased from Strandmøllen (Sweden). Glycerol stock of *E. coli* BL21 strain adapted for M9 minimal media was obtained as described in literature^18^. Chemicals used to prepare Control media, including DL-Alanine, Glycine, disodium hydrogen phosphate (Na_2_HPO_4_.2H_2_O), monopotassium phosphate (KH_2_PO_4_), sodium chloride (NaCl), magnesium sulfate (MgSO_4_), calcium chloride (CaCl_2_), and ammonium chloride (NH_4_Cl), were obtained from Sigma-Aldrich (Schnelldorf, Germany). Pure water was prepared with a Milli-Q device from Millipore (Billerica, MA, USA). Vacuum filtration system with a 0.2 μm polyethersulfone (PES) membrane for bacteria media sterilization was purchased from VWR (Stockholm, Sweden). 10 kDa Amicon filters (0.5 mL) and 0.2 μm syringe filters were purchased from Millipore. Petri dishes (90 × 15 mm), inoculating loops and corning sterile culture tubes (16 × 125 mm) were purchased from Sigma-Aldrich. Sterile plastic conical tubes (50 mL and 15 mL) for sample preparation were purchased from Sarstedt (Nümbrecht, Germany). The BioScreen C automatic fermentor was obtained from Oy Growth Curves AB Ltd (Helsinki, Finland).

The Miller-Urey apparatus (Fig. S1) was assembled according to literature^19^. After evacuating the whole apparatus by the pump, all the outlets were closed and held sealed for 2 h to ensure absence of leakage. Then about 250 mL of distilled water was added into the boiling flask, and boiling was started to remove all gasses dissolved in water. After that, the system was cooled down and re-evacuated to remove the released gases. Then, hydrogen was added to 169 mbar pressure after evacuating the hydrogen gas line. Then the manifold and hydrogen line were re-evacuated. Similarly, methane was loaded to 508 mbar, and finally ammonia was loaded to 846 mbar. After the gas loading, the heater was turned on to make continuous and gentle boiling. After the pressure became stable, sparking was started with a minimum voltage to maintain the spark continuously. Samples of MU mixtures were taken from the U-shape of the apparatus every one or two days of abiotic synthesis and stored in a freezer for further use.

### GC/MS analysis

MU sample was split into two equal portions. The “accelerated water sample preparation - AWASP” method^20^ was used for extraction: 1 mL of dichloromethane was added to the first portion of the sample, followed by addition of ~0.5 g of sodium sulfate to bind water. The sample was vigorously shaken. The transparent dichloromethane phase was transferred to another vial (Sample 1a). The same procedure was repeated with the second half of the sample, while acetonitrile was used instead of dichloromethane as an organic solvent (Sample 2a).

For derivatization, 25 μL of MSTFA was added to Sample 1a and the vial was heated to 40 °C for 25 min. In Sample 1b, the solvent was completely removed in a SpeedVac, then 25 μL of MSTFA was added and the temperature of the reaction mixture was increased to 80 °C for 15 min. Sample 1b was then transferred to a GC vial with a 250 μL insert. All samples were subjected to identical GC/MS analysis. Chromatographic separation of the molecules was performed using an Rxi-5SilMS column 30 m × 0.25 mm (id) × 0.25 mm (df) (Restek Corporation, Bellefonte, PA) with a helium flow of 1 mL/min. All injection volumes were 2 μL, split 10:1. The septum purge flow was 3 mL/min. The temperature of the injector, transfer line and ion source was 250 °C. A 3 min solvent delay was imposed for all runs. Prior to all experiments, the source and instrument ion optics were tuned using FC-43. The oven program was as follows: 3 min isothermal at 30 °C, then 10 °C/min to 180 °C and then 12 min isothermal before cooling. High-resolution accurate-mass GC-MS data were obtained using a Pegasus GC-HRT time-of-flight mass spectrometer (LECO Corporation, St Joseph, MI, USA) coupled to an Agilent 7890 A Gas Chromatograph (Agilent, Palo Alto, CA, USA). The system was controlled by the ChromaTOF-HRT software version 1.90.33 (LECO Corporation), which was also used for data collection and data processing. The data were collected using 10 full (26–510 m/z range) spectra per second in high resolution mode (25,000 at FWHM). Mass spectra were searched using NIST14 mass spectral library containing 276,248 electron ionization mass spectra of 242,466 compounds. In all cases, the identified compounds represented the best hit; the accepted threshold score (Match factor) was 750. The molecular mass tolerance was ±5 ppm. Since the molecular masses of all identified compounds were below 200 Da, such mass accuracy provided unique elemental composition. Furthermore, matched experimental mass spectra were manually inspected for the presence of significant fragment ions.

### LC/MS amino acid analysis

Amino acid standards (*puriss p. a*.) were purchased from commercial suppliers. Solvent acetonitrile (HPLC grade) was obtained from Sigma-Aldrich and water was purified by in-house Milli-Q water purification system (Millipore, Bedford, MA, USA) with a resistance >18 MΩ.cm^−1^. Formic acid (≥98%) was obtained from Sigma-Aldrich. A 20 amino acid standard stock solution was made by dissolving individual L-amino acid standards (alanine, arginine, asparagine, aspartic acid, glutamic acid, glycine, glutamine, histidine, isoleucine, leucine, lysine, phenylalanine, proline, serine, threonine, tryptophan, tyrosine, valine, norvaline and alpha-aminobutyric acid) in Milli-Q water. The standards and MU samples were derivatized with 6-aminoquinolyl-*N*-hydroxysuccinimidyl carbamate (AQC) using the Waters AccQ•Tag kit (WAT052875, Waters, Milford, MA, USA). Briefly, 20 μL of standard/sample solution was buffered with 60 μL of 0.2 M borate, derivatized with 60 μL of AQC reagent solution, and centrifuged at 16,100 × g for 3 min to remove particles. The supernatant was dried with nitrogen gas and reconstituted into 30 μL of 5% acetonitrile in water. A 10-μL aliquot of this solution was injected into the column for ultra-high performance liquid chromatography coupled with tandem mass spectrometry (UHPLC-MS/MS) analysis. The UHPLC-MS/MS system consisted an Accela pump and Accela auto-sampler UHPLC system (Thermo Fisher Scientific, San Jose, USA) coupled with a TSQ Vantage triple quadrupole mass spectrometer (Thermo Fisher). UHPLC separation was carried out with an ACCQ-TAG^TM^ ULTRA C18 column (100 × 2.1 mm, 1.7 μm particle size, Waters, Ireland) and a binary mobile phase (solvent A: 5% acetonitrile in water with 0.1% formic acid; solvent B: acetonitrile with 0.1% formic acid) delivered at a flow rate of 200 μL/min. The linear gradient elution program was as follows: 0.0 min − 0% B; 10.0 min − 5% B; 25.0 min − 15% B; 40.0 min − 22.5% B; 40.1 min − 80% B; 43.0 min − 80% B; 43.1 min − 0% B, and 50.0 min − 0% B. AQC derivatives of analytes were analyzed in positive ion detection mode using electrospray ionization and selected reaction monitoring (SRM) scan type. In order to improve the ion transmission efficiency, SRM scan time in the beginning of 40 min of LC gradient was divided into four time segments. Different SRM transitions and scan times in each time segment are list in Table S2. Other MS instrument parameters were as follows: spray voltage (4000 V), vaporizer temperature (300 °C), capillary temperature (300 °C), sheath gas pressure (30 psi), ion sweep gas pressure (0 psi), auxiliary gas pressure (20 psi), S-lens (110 V), de-clustering voltage (−6 V) and argon collision gas pressure (1.0 mTorr), and collision energy (25 V).

### E. coli adaptation to Control media

5-time concentrated M9 minimal salts stock solution was prepared by dissolving 42.5 g Na_2_HPO_4_ . 2H_2_O, 15 g KH_2_PO_4_ and 2.5 g NaCl in Milli-Q water to a final volume of 1000 mL. The solution was then sterilized by autoclaving at 121 °C for 20 min and stored at 4 °C for further use. M9 minimal media were prepared by mixing the following components: 800 mL Milli-Q water, 200 mL M9 concentrated salts stock solution, 2 mL of 1 M MgSO_4_ solution, 0.1 mL of 1 M CaCl_2_ solution, 1 g NH_4_Cl, 5 g mixture of DL-alanine and glycine (molar ratio 1:1). *E. coli* previously adapted to M9 minimal media was inoculated into 5 mL Control media and cultured at 37 °C for about 24 h. The optical density (O.D.) of the overnight culture exceeded 1.0. Each day, 5 μL overnight *E. coli* culture was diluted 1000 times by 5 mL fresh Control media and cultured at 37 °C continuously. In this way, nine to ten generations were grown between dilutions. The process continued for around three months, and ca. 800 generations adapted to Control media were obtained.

### E. coli growth measurements

The MU mixture was sterilized by filtering through 0.2 μm membrane and further through a 10 kDa membrane filter before being used for bacteria growth. The overnight culture of *E. coli* adapted to Control media (O.D. ≈ 1.4) was diluted with Control media to reach O.D. ≈ 0.7. A 5 μL aliquot of the diluted *E. coli* culture (O.D. ≈ 0.7) was added into 35 mL Control media to obtain the diluted *E. coli* culture for sample preparation. The samples were prepared using a programmed robotic system (Tecan, Genesis RSP 150, Männedorf, Switzerland). The sample arrangements on the 100-well honeycomb plates are given in Figs S2–4.

The BL21 strain of *E. coli* adapted to grow in a M9 minimal media containing glucose as the only source of carbon^18^ was grown in a 100-well honeycomb plate designed for bacterial growth measurements. The plate contained multiple sample/standard pairs of wells filled with 270 (or 290) μL of Control media containing a 1:1 mixture of Gly and reacemized Ala as well as the same inorganic salts as in M9. To each of the sample well on the plate, 30 (or 10) μL of filtered MU mixture was added to the total volume of 300 μL, and to each control well—the same volume of either pure water or Control media (Fig. S2).

Growth rate parameters were extracted as described in literature^18^. Briefly, the logarithm of O.D. was plotted against time. The slope for every 8-h interval was calculated, and the maximum value was determined as the maximum growth rate. The extrapolation of the line with maximum slope to the background level of O.D. gave the lag time. The maximum O.D. for each replicate minus the background O.D. was taken as the maximum density. The p-values were calculated using two-tailed, paired Student’s t-test.

## Results and Discussion

### Direct E. coli growth in MU mixture

Usually, bacteria in a richer media exhibit shorter lag phase, faster maximum growth and achieve higher maximum density^18^. The first experiment with MU mixture yielded strikingly negative results. A small (≈3%) admixture of the MU mixture obtained after 5–8 days of MU experiment inhibited *E. coli* growth not only in sample wells, but also in control wells. The experiment was repeated three times, and each time no growth was observed even in the control wells containing no MU mixture. This result testifies to the high toxicity of the MU mixture, in agreement with the earlier concerns^14^. Apparently, toxic fumes spread from the sample wells across the 100-well plate. GC-MS analysis showed that the MU mixture contains such toxic components as hydrogen cyanide, cyanic acid, diethylamine, pyridine and allyl alcohol. Other dangerous compounds were also identified, e.g., methyl- and ethylamine, N,N-dimethylacetamide, formamide, butanedinitrile, and 1-methylpyrrolidinone-2 (Table S1). GC-MS also detected presence of glycine and alanine, while heavier amino acids were not amenable to this technique.

In the fourth experiment, when a lighter 3^rd^ day MU fraction was used, some growth was observed, but it was strongly delayed (Fig. S3). Even though the bacteria grown in sample wells eventually reached higher density than in the control wells, this result did not unequivocally verify the life-supporting properties of the MU mixture.

### Adaptation of E. coli to MU mixture

To address the above issues, we pursued two strategies. First, we adapted the bacteria to grow in a synthetic mixture containing glycine and racemic alanine in equal proportions. Second, we dried and reconstituted the MU mixture, thus largely removing its volatile toxic components. To reach the first goal, the adapted to M9 minimal media *E. coli* strain BL21 was grown on (Gly + DL-Ala)-mixture supplemented with inorganic salts for ca. 800 generations (three months with daily media changes and reseeding). The adaptation progress was monitored with a BioScreen C automatic fermentor using the accurate method of growth parameter measurements^18^. Adaptation led to a 16% reduction in the lag phase duration, a 67% increase in the maximum growth rate as well as a 63% increase in the maximum density.

**Figure 1:**
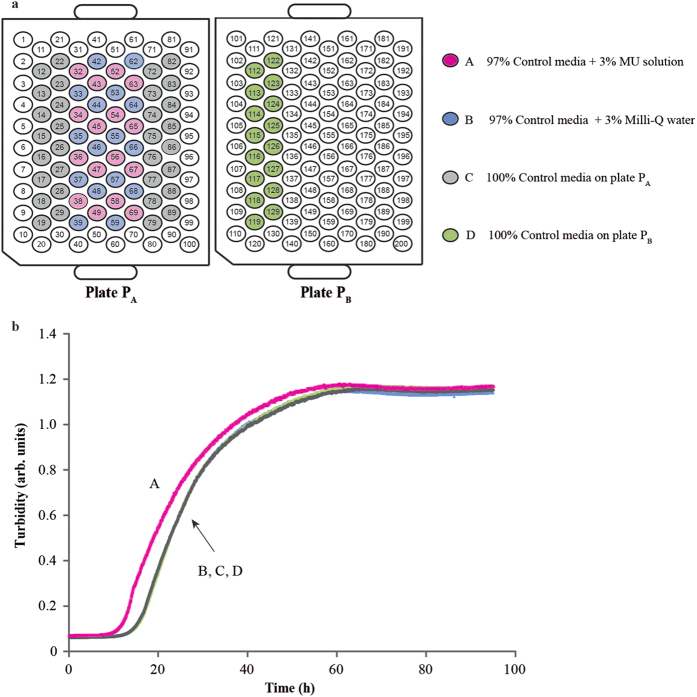
Growth experiment with *E. coli* adapted to control mixture. Top: sample layout. Bottom: examples of growth curves for different media: A − 97% Control mix + 3% reconstituted MU solution; B − 97% Control mix + 3% MiliQ water; C, D – 100% Control mix on two different 100-well plates.

Then the MU mixture was dried in a SpeedVac, the solid residue reconstituted in the same volume of pure water, and the growth experiment was conducted with (Gly + DL-Ala)-adapted bacteria. This time addition of MU mixture produced evident boost of growth compared not only to the wells where pure water was added, but also compared to the wells containing 100% Control media (Fig. 1).

**Figure 2:**
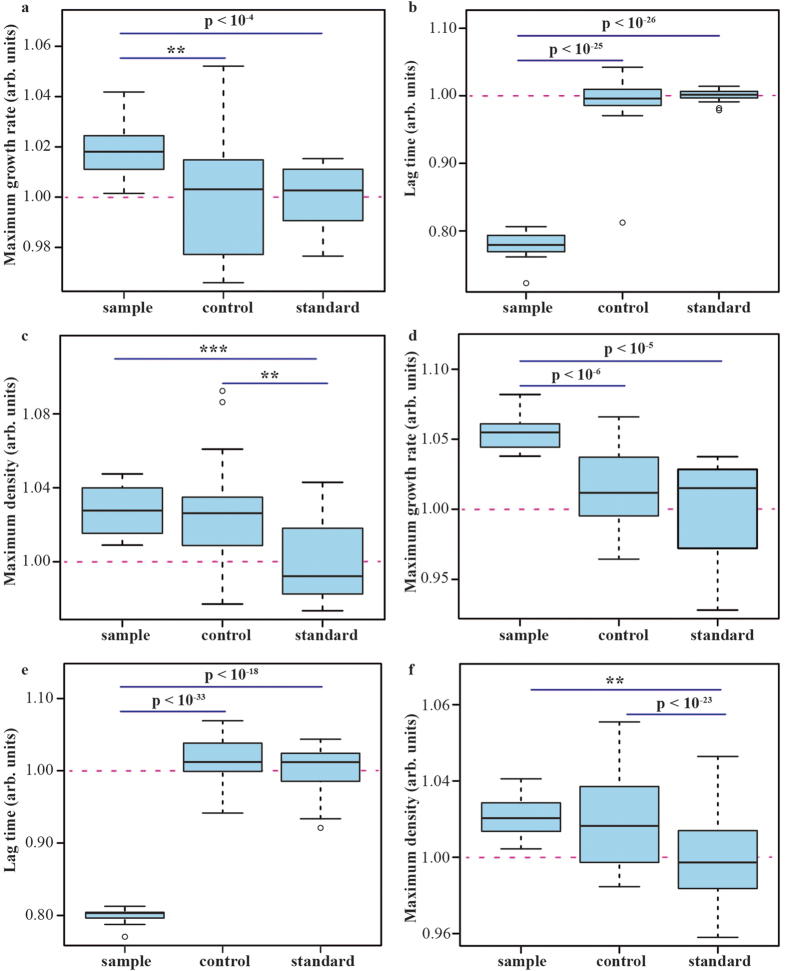
(a–c) Parameters of *E. coli* growth in a mixture of glycine and racemized alanine. Sample − 97% Control mix + 3% reconstituted MU solution; Control – 100% Control mix; Standard – 97% Control mix + 3% MiliQ water. (**d–f**) Results of a replicate experiment.

**Figure 3:**
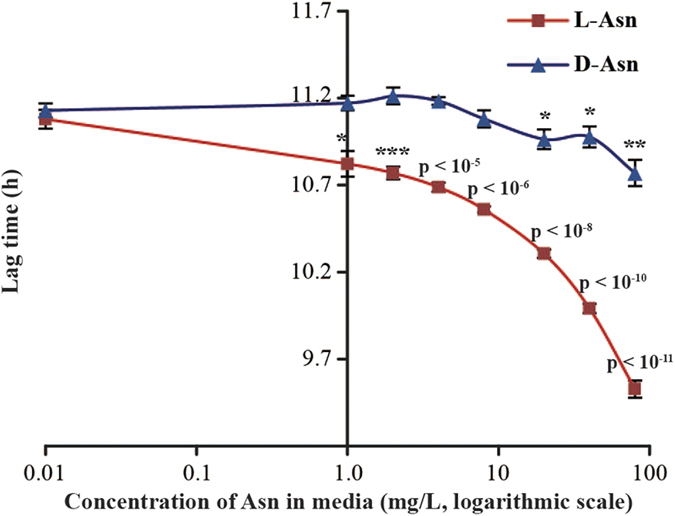
The decrease of the lag time in *E. coli* growth when either L- or D-Asn is added to the minimal media.

The experiment was repeated twice, with very similar results: faster growth was found in all three domains of measurements (Fig. 2). The strongest effect was a 20% reduction in the lag phase. This was not completely unexpected: while *E. coli* bacteria can grow on pure D-amino acids^17^, the presence of certain L-amino acids in the media can reduce their lag time significantly. Derivatization-assisted liquid chromatography separation of filtered MU mixture followed by mass spectrometric detection identified and quantified several biological amino acids. Besides the dominant Gly and Ala, Asn was detected (Table 1), in full agreement with previous studies^7^,^9^,^10^. In a separate experiment, addition of 1 mg/L of L-Asn to M9 minimal media reduced the lag time of *E. coli* as much as the addition of 80 mg/L of D-Asn (Fig. 3). This result confirmed that the presence of L-Asn was the likely cause of the growth acceleration effect of the dried MU mixture compared to Control media.

**Figure 4:**
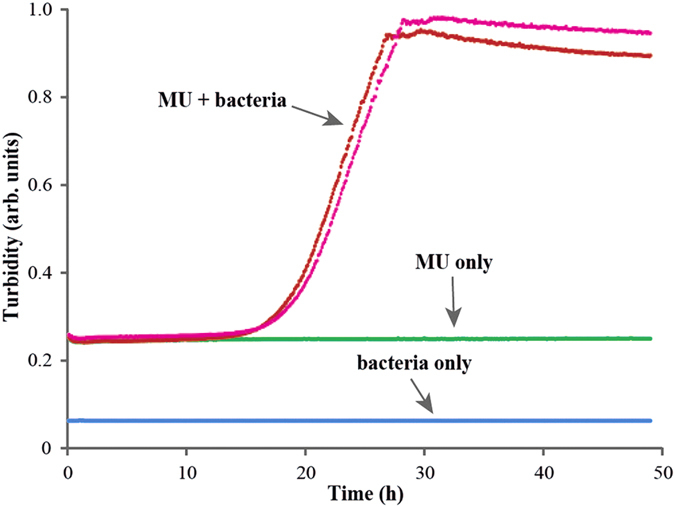
Growth curves for *E. coli* grown in dried and reconstituted in water MU mixture (red and pink), as well as controls: blue—bacteria in pure water and green—MU mixture without bacteria. Inorganic salts were added to all samples and controls.

In the last experiment, bacteria were made to grow in pure reconstituted MU mixture, with addition of only inorganic salts. Strong growth detected in two replicates (Fig. 4) leaves no doubt that dried and reconstituted primordial soup supports proliferation of *E. coli* adapted to a mixture of racemized simple amino acids.

## Conclusions

Our experiments demonstrate that adapted bacteria can grow on reconstituted MU mixture as the only source of carbon. Bacterial adaptation as well as abiotic mixture drying and reconstitution in our experiments closely mimic the corresponding processes that are presumed to occur naturally on the early Earth. Ancient bacteria had millions of years to adapt to the available sources of carbon. The “cleansed” prebiotic soup could accumulate in well-aired large and small water pools, providing food for early life. Thus, our planet provided a hospitable environment for early life forms, regardless whether they spontaneously emerged on Earth or have arrived from outer space. The presence of the isotopic resonance in terrestrial isotopic compositions^21^,^22^ was an additional advantage factor for early life.

It would be too simplistic to suggest that, because of the availability and even abundance on early Earth of accumulated prebiotically synthesized organic molecules, life must have originated on our planet from these compounds. Almost inevitably, early life would need to include rather unstable molecules that could not have accumulated in large quantities because of their short life time, such as RNAs. But it stands to reason that early metabolic machinery very quickly gained the ability to utilize the accumulated more stable compounds as a source of carbon and energy, simply because of their high abundance.

In interpreting the results of our study, it is important to remember that, on the one hand, modern organisms, such as *E. coli*, have arguably much more sophisticated metabolic machinery than primitive life forms. But on the other hand, the abundance of complex organic molecules in modern environment could have rendered the ability to process prebiotically synthesized simple organic compounds useless. Therefore, bacteria could have lost this ability in the process of evolution. Our experiment showed, however, that modern organisms can survive and thrive on a diet of prebiotically synthesized organic compounds. This ability might be the remnant of the early times when complex food was scarce on Earth. Therefore, early Earth would be a hospitable place for such organisms or their ancient counterparts, provided the latter had a similar metabolic competence.

If early life has come from other planets or star systems in a more or less developed form, as the panspermia hypothesis suggests, the abundance of primitive but edible food Earth provided for this early life might have been a decisive factor that determined its survival on our planet. One might also speculate that the type of easily available food had shaped the forms of life that have emerged and developed on Earth. However, there is currently not enough available knowledge to support such strong interpretation, in view of the RNA World and other competing hypotheses.

As a final comment, the Miller-Urey like synthesis analysed in this work was almost certainly complemented by the exogenous delivery of organics (e.g., kerogen-like polymers) by comets, meteorites and interplanetary dust particles entering Earth atmosphere. It is currently unknown whether this type of “imported” organics was edible. Further experiments are needed to clarify this issue.

## Additional Information

**How to cite this article**: Xie, X. *et al.* Primordial soup was edible: abiotically produced Miller-Urey mixture supports bacterial growth. *Sci. Rep.* **5**, 14338; doi: 10.1038/srep14338 (2015).

## Supplementary Material

Supplementary Information

## Figures and Tables

**Table 1:** Concentrations of amino acids found in the filtered MU mixture.

Amino Acids	Conc., ppm	Conc., %
	(n = 3)	(n = 3)
Gly	10.9 ± 0.6	55.4 ± 3.2
Ala	5.7 ± 0.4	28.9 ± 2.2
Abu	0.16 ± 0.04	0.8 ± 0.2
Ser	0.76 ± 0.04	3.9 ± 0.2
Pro	0.01 ± <0.01	0.07 ± <0.01
Val	0.01 ± <0.01	0.03 ± <0.01
NoVal	0.08 ± <0.01	0.39 ± 0.01
Thr	0.05 ± <0.01	0.23 ± 0.01
Asn	1.25 ± 0.02	6.4 ± 0.1
Asp	0.71 ± 0.04	3.6 ± 0.2
Glu	0.04 ± <0.01	0.19 ± 0.01
His	0.004 ± <0.001	0.02 ± <0.01
Arg	0.005 ± <0.001	0.02 ± <0.01
Trp	0.001 ± <0.001	0.004 ± <0.001
**Sum**	**19.68**	**100**

Abu—aminobutyric acid, NoVal—norvaline.
