# Supplementary material for: Primordial soup was edible: abiotically produced Miller-Urey mixture supports bacterial growth
Source: Sci Rep. 2015 Sep 28;5:14338. doi: 10.1038/srep14338 (PMC4585927; doi:10.1038/srep14338)
Supplement: Supplementary Information [file srep14338-s1.pdf]

Primordial soup was edible: abiotically produced Miller-Urey mixture supports bacterial growth

Supplementary Materials

*Xueshu Xie[1], Daniel Backman[1], Albert T. Lebedev[2], Viatcheslav B. Artaev[3], Liying Jiang[4], Leopold L. Ilag[4], and Roman A. Zubarev[1]**

[1]Division of Physiological Chemistry I, Department of Medical Biochemistry and Biophysics, Karolinska Institutet, Scheelesväg 2, SE-17 177 Stockholm, Sweden

[2]Department of Organic Chemistry, Moscow State M. V. Lomonosov University, 119991, Moscow, Russia.

[3]LECO Corporation, 3000 Lakeview Avenue, St Joseph, MI, USA.

[4]Department of Analytical Chemistry, University of Stockholm, Stockholm, Sweden.

*Corresponding author: Roman.Zubarev@ki.se

Sample preparation for *E. coli* growth measurements

The MU mixture was sterilized by filtering through 0.2 µm membrane and further through a 10 kDa membrane filter before being used for bacteria growth. The overnight culture of *E. coli* adapted to Control media (O.D. ≈ 1.4) was diluted with Control media into O.D. ≈ 0.7.

Experiment one: Admixture of MU mixture obtained after 5-8 days of MU experiment was used for this experiment. A 5 µL aliquot of *E. coli* culture (O.D. ≈ 0.7) was added into 35 mL Control media and mixed well to obtain the diluted *E. coli* culture for later sample preparation. First, 10 µL aliquot of pure Control media were added into each of the E wells on the left side of the plate (marked as grey in Figure S2) and 30 µL aliquot of pure Control media were added into each of the E wells on the right side of the plate (marked as grey in Figure S2) to serve as control. Second, 10 µL aliquot of sterile and filtered MU mixture was added into each of the A wells (marked as pink in Figure S2) and 30 µL of the MU mixture was added into each of the C wells (marked as red in Figure S2) by pipette. Third, 10 µL aliquot of sterile Milli-Q water was added into each of the B wells (marked as blue in Figure S2) and 30 uL aliquot of Milli-Q water was added into each of the D wells (marked with light green in Figure S2). Forth, 300 µL sterile Milli-Q water were add with a programmed robotic system (Tecan, Genesis RSP 150, Männedorf, Switzerland) into each of the border wells ("edge cells") on the plate (no color code in Figure S2). Finally, 290 µL of the diluted *E. coli* culture (35 mL) was dispensed into each of the A wells, B wells and left-side E wells and 270 µL of the same diluted *E. coli* culture was dispensed into each of the C wells, D wells and right-side E wells by using the Tecan robot.

After sample preparation, the well plate was placed into the Bioscreen C instrument and *E. coli* was grown with continuous shaking at 37 ^{o}C. Turbidity of *E. coli* in each well was measured (with wide band filter 420–580 nm) every six minutes by Bioscreen C instrument to obtain a raw growth curve.

Experiment two: Repeat of Experiment 1.

Experiment three: Repeat of Experiment 1.

Experiment four: A 3rd day of MU fraction was used for this experiment. A 5 µL aliquot of *E. coli* culture (O.D. ≈ 0.7) was added into 35 mL Control media and mixed well to obtain the diluted *E. coli* culture for later sample preparation. First, 10 µL aliquot of pure Control media were added into each of the C wells on the plate A (marked as grey in Figure S3) as well as each of the D wells on the plate D (marked as green in Figure S3) by pipette to serve as control. Second, 10 µL aliquot of sterile and filtered MU mixture was added into each of the A wells (marked as pink in Figure S3) to prepare 16 replicates of sample. Third, 10 µL aliquot of sterile Milli-Q water was added into each of the B wells (marked as blue in Figure S3) to prepare 16 replicates of standard. Forth, 300 µL sterile Milli-Q water were add with a programmed robotic system (Tecan, Genesis RSP 150, Männedorf, Switzerland) into each of the border wells (“edge cells”) on the both plate A and plate B (no color code in Figure S3). Finally, 290 µL of the diluted *E. coli* culture (35 mL) was dispensed into each of the wells A, B, C, D by using the Tecan robot.

After sample preparation, the well plate was placed into the Bioscreen C instrument and *E. coli* was grown with continuous shaking at 37 ^{o}C. Turbidity of *E. coli* in each well was measured (with wide band filter 420–580 nm) every six minutes by Bioscreen C instrument to obtain a raw growth curve.

Experiment five: Repeat Experiment 4 with the dried and reconstituted MU mixture. Sample layout was as shown in Figure 1. The MU mixture used for this experiment was first dried by using SpeedVac to release all volatile compounds. The completely dried MU mixture was then reconstituted by Milli-Q water to have the same final volume as before being dried. The dried and reconstituted MU mixture was sterilized by filtration before being used for this experiment.

Experiment six: Repeat of Experiment five.

Experiment seven: Inorganic salts solution was prepared with all components as the Control media except glucose. The completely dried MU mixture was then reconstituted with the prepared inorganic salts solution. The dried and reconstituted MU mixture was sterilized by filtration before being used for this experiment. A 65 µL aliquot of *E. coli* culture (O.D. ≈ 0.3) was added into 15 mL Inorganic salts solution and mixed well to obtain *E. coli* inorganic salts solution for later sample preparation. 280 µL aliquot of dried and reconstituted MU mixture into each of the A (marked as pink in Figure S4) and B wells (marked as light green in Figure S4). And 280 µL aliquot of pure inorganic salts solution into each of C wells (marked as blue in Figure S4). Then 20 µL aliquot of the *E. coli* inorganic salts solution was added into each of A and C wells while 20 µL aliquot of pure inorganic salts solution without bacteria was added into each of the B wells.

After sample preparation, the well plate was placed into the Bioscreen C instrument and *E. coli* was grown with continuous shaking at 37 ^{o}C. Turbidity of *E. coli* in each well was measured (with wide band filter 420–580 nm) every six minutes by Bioscreen C instrument to obtain a raw growth curve.

L-Asn and D-Asn effect on *E.coli* growth

21.05 mg L-Asn (132.12 g/mol) and 19.26 mg D-Asn (monohydrate, 150.13 g/mol) were weighed and dissolved in M9 media to obtain 12,000 mg/L L-Asn and D-Asn stock solution. The two stock solutions were then diluted 10 times by mixing 700 µL stock solution with 6300 µL M9 media to make 1,200 mg/L L- and D-Asn solution followed by sterilization by filtration.

Two series solution of L-Asn (0, 15, 30, 60, 120, 300, 600, 1200 mg/L) and D-Asn (0, 15, 30, 60, 120, 300, 600, 1200 mg/L) were prepared by diluting the two stock solution of L-Asn or D-Asn with M9 minimal media for further use.

A 6.7 µL aliquot of *E. coli* culture (O.D. ≈ 0.6) was added into 45 mL M9 media and mixed well to obtain the diluted *E. coli* culture for later sample preparation.

All edged wells on both Plate A and Plate B were filled with 300 µL M9 media to serve as blanks (same positions as Figure S2). Each 20 µL aliquot of L-Asn solution were added on each well on Plate A except edged wells to prepare 8 replicates of sample with different concentrations of L-Asn (0, 1, 2, 4, 8, 20, 40, 80 mg/L) as the following: 0 mg/L L-Asn to each well of second column; 15 mg/L L-Asn to each well of third column; 30 mg/L L-Asn to each well of forth column; 60 mg/L L-Asn to each well of fifth column; 120 mg/L L-Asn to each well of sixth column; 300 mg/L L-Asn to each well of seventh column; 600 mg/L L-Asn to each well of eighth column; 1200 mg/L L-Asn to each well of ninth column. The series of D-Asn solution were added onto the Plate B in the same way as L-Asn.

Finally, 280 uL of the diluted *E. coli* culture (45 mL) was added into each well on both plate A and plate B except the edged wells (blanks).

After sample preparation, the well plate was placed into the Bioscreen C instrument and *E. coli* was grown with continuous shaking at 37 ^{o}C. Turbidity of *E. coli* in each well was measured (with wide band filter 420–580 nm) every six minutes by Bioscreen C instrument to obtain a raw growth curve.

Supplementary Tables

Table S1. Compounds determined in MU mixture by GC-MS analysis.

Toxic Compounds	Dangerous Compounds	Other Compounds
Hydrogen cyanide	Methylamine	N-ethyl-N-methylethanamine
Cyanic (isocyanic) acid	Ethylamine	Ethanol
Diethylamine	N,N-dimethylacetamide	Isopropanol
Hyridine	Formamide	N-methylformamide
Allyl alcohol	Butanedinitrile	Acetamide
	1-methylpyrrolidinone-2	Propanamide
		Propanediamide
		Aminoacetonitrile
		2-Aminopropanenitrile
		N-methylaminoacetonitrile
		2-Ethylideneaminoacetonitrile
		2-Ethylideneaminopropionitrile
		4,5-Dihydro-5-methyl-1H-pyrazole
		Glycerol
		Formic acid
		1,2-Propanediol
		2-Hydroxypropanoic acid
		Hydroxyacetic acid
		Alanine
		Glycine
		Urea

Table S2: SRM transitions and scan times in 4 time segments used in UHPLC-MS/MS method for AQC-derivatives of amino acids.

	LC duration (min)	Amino acids (Abbreviation)	SRM transition (m/z)	Scan time (ms)
**Segment 1**	2.10	Histidine (His)	326.13>171.06	500
**Segment 2**	4.70	Glycine (Gly)	246.09>171.06	100
		Serine (Ser)	276.10>171.06	100
		Threonine (Thr)	290.11>171.06	100
		Asparagine (Asn)	303.11>171.06	100
		Aspartie acid (Asp)	304.09>171.06	100
		Glutamine (Gln)	317.12>171.06	100
		Glutamic acid (Glu)	318.11>171.06	100
		Arginine (Arg)	345.17>171.06	300
**Segment 3**	18.00	Alanine (Ala)	260.10>171.06	100
		alpha-aminobutyric acid (Abu)	274.12>171.06	100
		Proline (Pro)	286.12>171.06	100
		Valine (Val) or Norvaline (Nor-Val)	288.13>171.06	100
		Lysine (Lys)	317.16>171.06	500
		Tyrosine (Tyr)	352.13>171.06	100
**Segment 4**	15.20	Leucine (Leu) or Isoleucine (Ile)	302.15>171.06	100
		Phenylalanine (Phe)	336.13>171.06	100
		Tryptophan (Trp)	375.15>171.06	100

Supplementary Figures

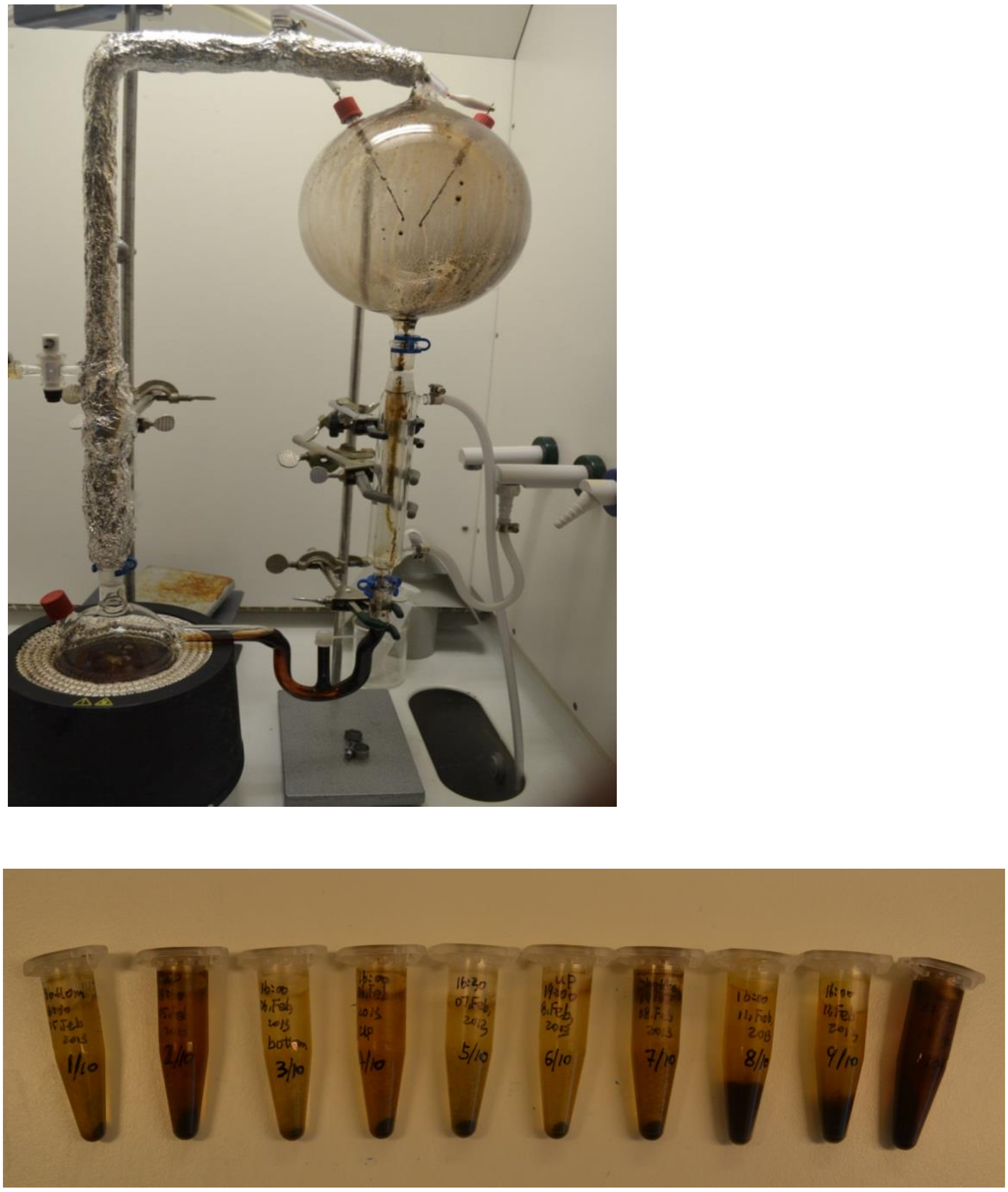

Figure S1. Top: Miller-Urey apparatus. Bottom: vials with aliquots taken from the MU apparatus after 1 to 10 days of synthesis.

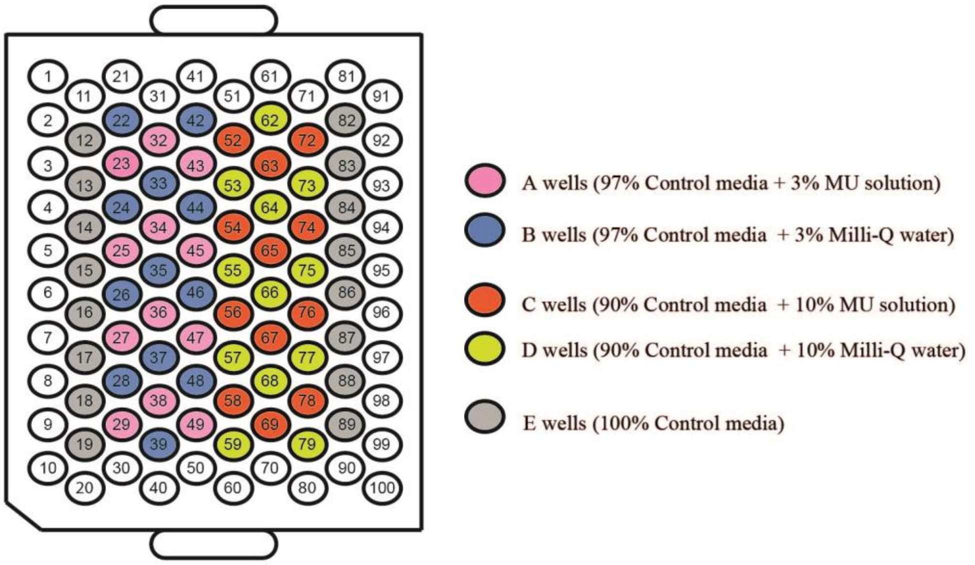

Figure S2. The layout of the 100-well plate of the BioScreen C automated fermentor. To each colored well containing 270 µL of Control media with adapted bacteria, 30 µL were added of either filtered MU mixture (wells A, C) or pure water as a control (wells B, D). As an additional control, bacteria were grown in 300 µL of Control media (wells E).

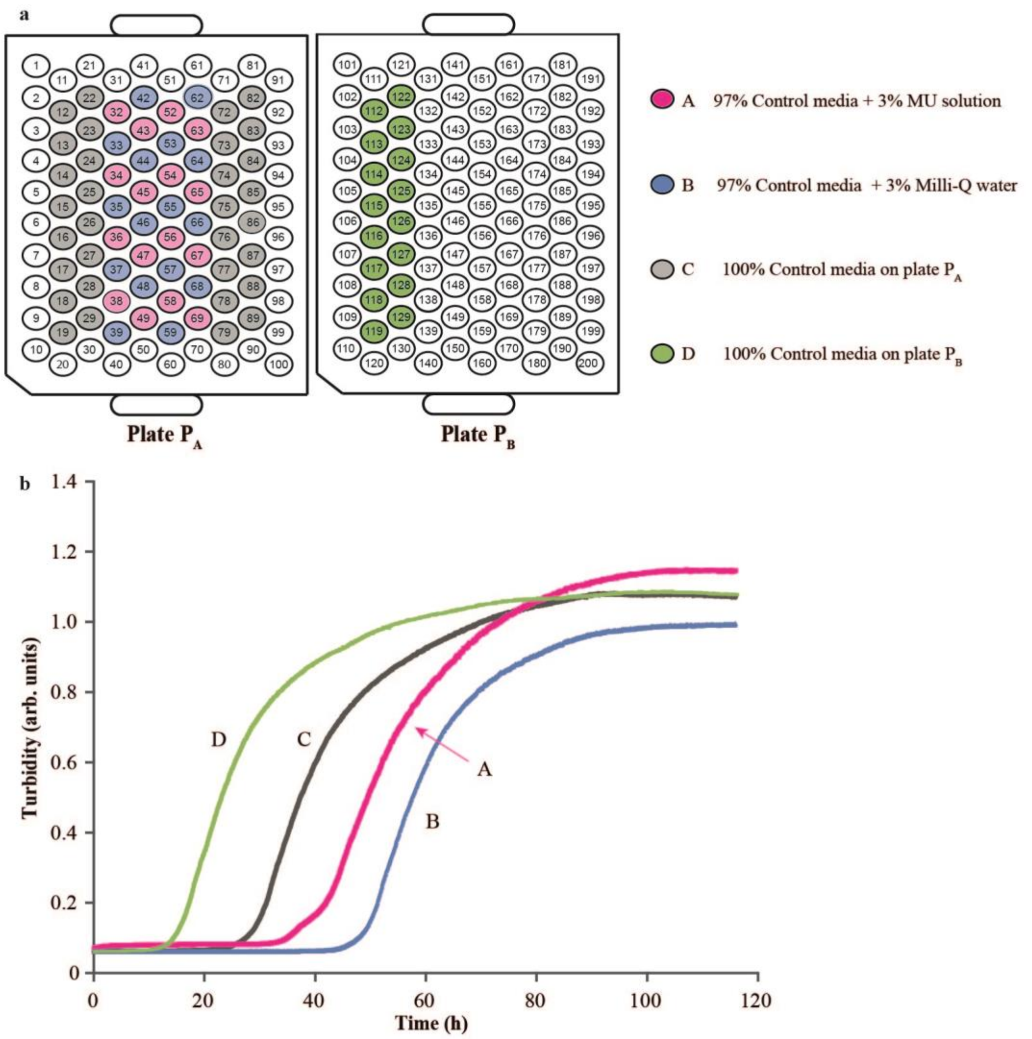

Figure S3. Experiment 4 when early MU fraction was used. (a) Sample layout. (b) Growth curves.

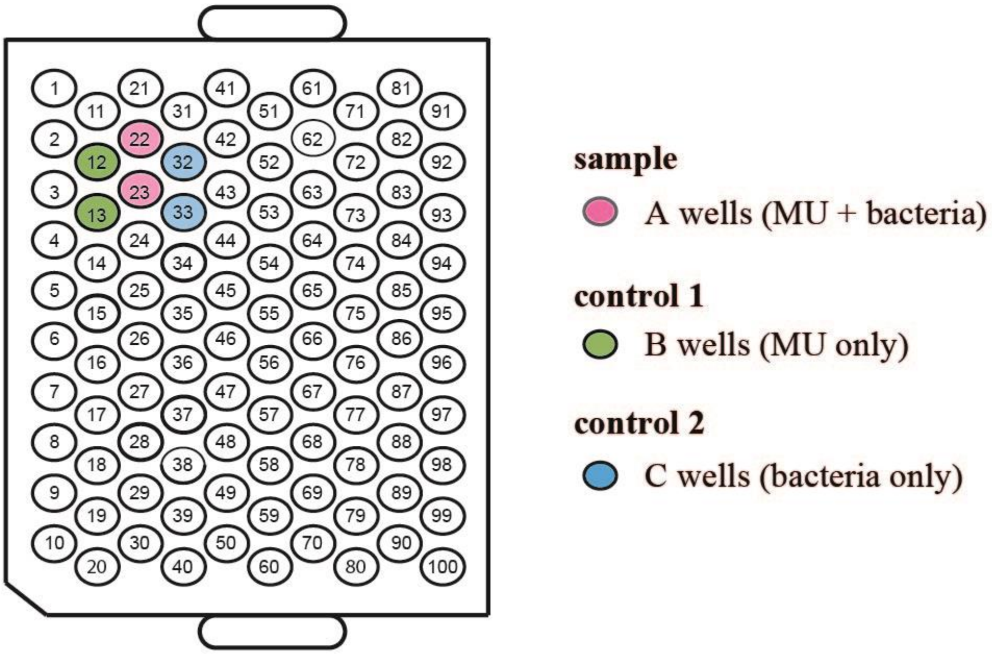

Figure S4. Sample layout of the experiment (Experiment seven) in Figure 4.
